# Early rehabilitation for critically ill adult patients: protocol for an umbrella review

**DOI:** 10.3389/fresc.2026.1885510

**Published:** 2026-07-22

**Authors:** Kun Sun, Yongming Tian, Yuanyuan Song, Biantong Jiang, Huan Liu, Aiping Du

**Affiliations:** 1 Department of Critical Care Medicine, West China Hospital, Sichuan University, Chengdu, Sichuan, China; 2 West China School of Nursing, Sichuan University, Chengdu, Sichuan, China

**Keywords:** critical care rehabilitation, critical illness, early rehabilitation, evidence synthesis, intensive care unit, patient safety, post-intensive care syndrome, umbrella review

## Abstract

**Background:**

Advances in critical care medicine have improved short-term survival for intensive care unit (ICU) patients, yet a large proportion of survivors experience lasting physical, cognitive, and psychological impairments collectively termed post-intensive care syndrome (PICS). Early rehabilitation (ER) is a core strategy to reduce PICS burden, but available systematic reviews (SRs) vary widely in intervention protocols, population scope, and methodological rigor. Evidence remains inconsistent regarding ER safety and efficacy in high-risk subgroups, and no high-level evidence synthesis has comprehensively appraised the current evidence base.

**Objective:**

This protocol outlines an umbrella review (overview of systematic reviews) designed to synthesise evidence on the effectiveness and safety of ER in critically ill adult patients, evaluate the methodological quality of existing SRs, and grade the certainty of evidence for key outcomes.

**Methods:**

Nine electronic databases (CINAHL, Cochrane Library, Embase, PubMed, Web of Science, CNKI, CBM, Wanfang, VIP) will be searched from inception to 30 April 2026, supplemented by manual reference screening and PROSPERO registry searches. Two independent reviewers will conduct title/abstract screening and full-text eligibility assessment, with disagreements resolved via consensus or third-party adjudication. Methodological quality of included SRs will be appraised using the AMSTAR 2 tool, and the certainty of evidence for primary outcomes will be graded using the GRADE framework. A narrative evidence synthesis will be performed to map findings across reviews; quantitative meta-analysis will only be conducted at the primary study level where methodologically appropriate, to avoid bias from overlapping study samples. Subgroup and sensitivity analyses will be undertaken to explore sources of heterogeneity.

**Anticipated contribution:**

This umbrella review will provide a rigorous, high-level synthesis of the current ER evidence base. If evidence certainty is sufficient, findings may inform evidence-based clinical decision-making, support standardised protocol development, and identify priority evidence gaps for future primary research.

**Systematic Review Registration:**

https://www.crd.york.ac.uk/PROSPERO/view/CRD420250651183, Registration No. CRD420250651183.

## 1 Introduction

Over the past two decades, advances in critical care medicine have substantially improved short-term survival among intensive care unit (ICU) patients, transforming many previously fatal conditions into survivable illnesses. As survival rates have risen, the priority of critical care has shifted beyond mere life support to optimising long-term functional recovery and health-related quality of life. A large and growing body of evidence demonstrates that ICU survivors frequently develop persistent physical, cognitive, and psychological impairments collectively termed post-intensive care syndrome (PICS). Systematic reviews indicate that PICS affects approximately 60% of patients at 3 months post-ICU discharge and 40% at 12 months, with manifestations including ICU-acquired weakness (ICU-AW), executive dysfunction, anxiety, depression, and post-traumatic stress disorder that may persist for years after hospital discharge (1). These impairments not only reduce patients' ability to return to independent living and employment but also drive substantial increases in post-discharge healthcare utilisation and long-term societal costs.

Early rehabilitation (ER) is a multidisciplinary, patient-centred intervention delivered by collaborative teams including intensivists, critical care nurses, respiratory therapists, physiotherapists, occupational therapists, and psychologists. For the purpose of this review, ER is explicitly defined as structured rehabilitation initiated within 72 h of ICU admission or 24 h of haemodynamic stabilisation (2), encompassing four core domains: physical rehabilitation, respiratory rehabilitation, cognitive rehabilitation, and psychological rehabilitation, aligned with international multimodal rehabilitation frameworks (3, 4). International clinical practice guidelines, including the 2018 Pain, Agitation/Sedation, Delirium, Immobility, and Sleep (PADIS) guidelines from the American College of Critical Care Medicine (ACCM), endorse early rehabilitation as a standard component of high-quality ICU care for haemodynamically stable patients, with initiation recommended as soon as clinically feasible within the first 72 h of admission (5, 6). Accumulating evidence from primary studies and systematic reviews confirms that ER can improve physical function, reduce delirium duration, shorten mechanical ventilation time and ICU length of stay, and enhance long-term quality of life (7–10).

Despite these established benefits, the existing evidence base for ER in critical illness has important limitations. First, there is marked heterogeneity across studies in ER protocols, including wide variation in initiation timing, intervention intensity, session duration, and component modalities, which limits comparability of findings and clinical generalisability. Second, most available systematic reviews (SRs) have focused predominantly on physical mobilisation in general ICU populations, while evidence regarding the efficacy and safety of ER in high-risk subgroups—such as patients receiving extracorporeal membrane oxygenation (ECMO), those with severe neurological injury, or older adults with pre-existing frailty—remains sparse and inconsistent. Third, adverse event reporting across primary trials and SRs is highly variable and incomplete, leaving the safety profile of ER insufficiently characterised. Fourth, methodological quality assessments of published systematic reviews in this field consistently demonstrate widespread shortcomings in study conduct and reporting, with fewer than 30% of available reviews meeting high-quality standards when evaluated using the AMSTAR 2 tool (11). Finally, overlapping primary study samples across published SRs may introduce bias and distort effect estimates, further weakening the reliability of available conclusions.

An umbrella review (also termed an overview of systematic reviews) is a high-level evidence synthesis methodology that systematically identifies, appraises, and integrates all relevant SRs on a given clinical topic (12). This approach addresses the limitations of individual SRs by mapping the full evidence landscape, quantifying methodological quality, identifying inconsistencies across reviews, grading the certainty of evidence, and clarifying where evidence gaps remain. While a 2024 scoping review mapped the volume and scope of existing SRs in this field, it did not perform formal methodological quality appraisal, evidence grading, or targeted synthesis of safety outcomes. The present overview builds on this prior work by: (1) applying the AMSTAR 2 tool to rigorously evaluate the methodological quality of all included SRs; (2) grading the certainty of evidence for core outcomes using the GRADE framework; (3) systematically synthesising evidence on ER safety and adverse events; (4) including both English and Chinese language SRs to provide a more global evidence base; and (5) exploring evidence for high-risk patient subgroups that have been underrepresented in prior syntheses. To date, to our knowledge, no comprehensive umbrella review with these features has been conducted for ER in critically ill adult patients.

Therefore, the primary objective of this study is to conduct a systematic umbrella review to evaluate the effectiveness and safety of ER in critically ill adult patients. Secondary objectives include: (1) comparing the efficacy of different ER protocols across timing, intensity, and modality; (2) assessing ER outcomes in high-risk patient subgroups; (3) evaluating the methodological quality of existing SRs in this field; and (4) identifying key evidence gaps to guide future primary research.

## 2 Methods

This protocol is reported in accordance with the Preferred Reporting Items for Systematic Review and Meta-Analysis Protocols (PRISMA-P) 2015 statement, the PRISMA for Overviews of Systematic Reviews (PRISMA-O) 2021 guidelines, and the PRIOR (Preferred Reporting Items for Overviews of Reviews) statement to ensure methodological rigor and transparent reporting.

### 2.1 Study design, registration and reporting guidelines

This manuscript presents the protocol for an umbrella review (overview of systematic reviews) evaluating the effectiveness and safety of early rehabilitation in critically ill adult patients. The term “umbrella review” is used consistently throughout the manuscript as the primary descriptor of the study design.

This protocol is reported in accordance with the Preferred Reporting Items for Systematic Review and Meta-Analysis Protocols (PRISMA-P) 2015 statement and the Preferred Reporting Items for Overviews of Systematic Reviews (PRISMA-O) 2021 guidelines. We additionally follow the PRIOR (Preferred Reporting Items for Overviews of Reviews) statement, the dedicated reporting framework for overviews of healthcare intervention reviews, to ensure methodological rigor and transparent reporting.

This protocol was prospectively registered in PROSPERO on 15 October 2025 (Registration No. CRD420250651183). All eligibility criteria, outcome measures, and analytical plans presented in this manuscript are fully aligned with the PROSPERO registration record. Any future amendments will be formally updated in the registry and explicitly documented in the final publication.

### 2.2 Eligibility criteria

#### 2.2.1 Inclusion criteria

- **Study type:** Published systematic reviews (SRs) or meta-analyses (MAs) evaluating early rehabilitation interventions in critically ill patients. Reviews synthesizing randomized controlled trials (RCTs), non-randomized controlled trials (NRCTs), or prospective observational cohort studies are all eligible. Reviews with mixed study designs will be included in full. Evidence from RCTs will form the primary basis for effectiveness outcome synthesis, while observational study data will be used exclusively to inform safety profiles and implementation feasibility.
- **Participants:** Critically ill adult patients (≥18 years) admitted to a formal intensive care unit (ICU, including general medical/surgical ICU, cardiac ICU, and neuro-ICU) for ≥24 h, meeting standard critical illness definitions (requiring organ support or continuous hemodynamic monitoring). Patients in step-down units, high-dependency units, or post-ICU ward rehabilitation settings are not eligible.
- **Interventions:** Any structured early rehabilitation (ER) intervention initiated within 72 h of ICU admission or within 24 h of hemodynamic stabilization. This time threshold is aligned with international critical care rehabilitation guidelines and consensus definitions of early mobilization (2, 5). ER encompasses four core domains:
  1. Physical rehabilitation (passive range-of-motion exercises, active resistance training, early ambulation)
  2. Respiratory rehabilitation (inspiratory muscle training, airway clearance techniques, breathing exercises)
  3. Cognitive rehabilitation (orientation training, cognitive stimulation)
  4. Psychological rehabilitation (anxiety management, relaxation therapy)

Reviews using alternative timing thresholds for “early” rehabilitation will not be excluded; their intervention timing will be extracted and stratified for subgroup analysis. We will also systematically extract data on delivery team composition (physiotherapist-led, nurse-led, multidisciplinary team) for subgroup comparison. Passive and active interventions will be analyzed separately, as they differ substantially in clinical indication, resource requirements, and expected effects.

- **Comparators:** Any control condition, including standard ICU care without structured ER, delayed rehabilitation, lower-intensity rehabilitation, or alternative ER protocols. Comparator groups will be stratified by type during synthesis to ensure valid, like-for-like comparisons.
- **Outcomes:** Studies reporting at least one prespecified primary or secondary outcome are eligible.

#### 2.2.2 Exclusion criteria

- SR protocols, conference abstracts, letters, commentaries, editorials, narrative reviews, and scoping reviews
- SRs exclusively enrolling pediatric patients (<18 years)
- SRs with incomplete data or unavailable full text
- Duplicate publications (only the most recent and methodologically comprehensive version will be retained)
- SRs not published in English or Chinese

Note: SRs focused on specific high-risk subgroups (e.g., ECMO recipients, severe traumatic brain injury, mechanically ventilated patients) or single rehabilitation modalities (e.g., respiratory muscle training) are not excluded. These reviews will be included and analyzed in targeted subgroup analyses.

#### 2.2.3 Outcome measures

All outcomes will be stratified by assessment time point (ICU discharge, hospital discharge, 3 months post-discharge, 6 + months post-discharge) and will not be pooled across time points.

- **Primary outcomes:**
  1. Physical function (assessed via MRC sum score, 6-minute walk distance, ADL score, or equivalent tools)
  2. Cognitive function (assessed via MMSE, MoCA, or equivalent tools)
  3. Psychological function (assessed via HADS, IES-R, or equivalent tools)

Note: Cognitive and psychological outcomes are designated as primary *a priori*. If reporting is highly inconsistent across included reviews, these outcomes will be reclassified as secondary exploratory outcomes and analyzed via narrative synthesis only.

- **Secondary outcomes:**
  1. ICU length of stay (LOS)
  2. Hospital LOS
  3. Duration of mechanical ventilation
  4. 28-day and 90-day all-cause mortality
  5. ICU readmission rate
- **Safety outcomes:**

Adverse events will be classified using a standardized taxonomy and counted at the patient level:

1. Severe adverse events: hemodynamic instability requiring intervention, new-onset arrhythmia, invasive line dislodgement, respiratory failure requiring escalation of support
2. Minor adverse events: falls without injury, transient desaturation, self-limited fatigue

### 2.3 Information sources and search strategy

#### 2.3.1 Databases searched

A comprehensive systematic search will be conducted across 9 electronic databases from inception to 30 April 2026:

- English databases: CINAHL (EBSCOhost), Cochrane Library, Embase (Ovid), PubMed, Web of Science Core Collection
- Chinese databases: China National Knowledge Infrastructure (CNKI), Chinese Biomedical Literature Database (CBM), Wanfang Database, VIP Database

Supplementary searches will include manual screening of reference lists of all included SRs and landmark reviews, as well as searching the PROSPERO registry to identify completed but unpublished SRs.

#### 2.3.2 Eligibility restrictions

- **Language:** Restricted to English and Chinese, consistent with the review team's language expertise
- **Publication type**: Peer-reviewed, full-text published SRs and MAs; conference abstracts, preprints, and uncompleted protocols are excluded
- **Population**: Human studies only

#### 2.3.3 Search strategy development

The search strategy combines controlled vocabulary (MeSH/Emtree terms) and extensive free-text synonyms, adapted to the syntax of each database. The strategy was developed in consultation with an academic health sciences librarian and will undergo formal peer review using the PRESS (Peer Review of Electronic Search Strategies) checklist prior to execution.

The revised PubMed search strategy is presented in Table 1. Full, database-specific search strategies will be provided as online supplementary material.

**Table 1:** Search strategy for PubMed.

Serial number	Search term
#1	“Rehabilitation”[Mesh] OR “Exercise Therapy”[Mesh] OR “Early Ambulation”[Mesh] OR “Physical Therapy Modalities”[Mesh] OR “Respiratory Therapy”[Mesh]
#2	(early rehabilitat* OR early mobil* OR early mobility OR early exercise* OR early activ* OR ambulation OR mobilization OR physiotherapy OR physical therapy OR rehabilitation therap* OR exercise therap* OR cardiopulmonary rehabilitat*).ti,ab.
#3	“Critical Illness”[Mesh] OR “Intensive Care Units”[Mesh] OR “Critical Care”[Mesh]
#4	(critical ill* OR intensive care OR ICU OR critical care unit* OR mechanically ventilat* OR post intensive care syndrome OR PICS).ti,ab.
#5	“Systematic Review”[pt] OR “Meta-Analysis”[pt]
#6	(systematic review* OR meta-analys* OR umbrella review* OR overview of review* OR review of reviews OR evidence synthesis).ti,ab.
#7	#1 OR #2
#8	#3 OR #4
#9	#5 OR #6
#10	#7 AND #8 AND #9
#11	Filters: Humans, English, Chinese

### 2.4 Study selection and data extraction

#### 2.4.1 Study selection process

All records will be imported into EndNote 21 for automated duplicate removal, then uploaded to Rayyan QCRI for blinded title/abstract screening and full-text review. Records from English and Chinese databases will be merged and managed in a single unified screening workflow.

Two independent reviewers (KS and BJ) will conduct selection in two sequential phases:

1. **Title/abstract screening:** Reviewers independently screen all records to exclude clearly ineligible studies. Records deemed potentially eligible by either reviewer proceed to full-text screening.
2. **Full-text eligibility screening:** Reviewers independently assess full-text articles against the eligibility criteria.

Disagreements are resolved by consensus discussion; if unresolved, a third senior reviewer (YT) adjudicates. Inter-rater reliability is calculated using Cohen's kappa coefficient, with *κ* ≥ 0.8 defined as excellent agreement.

**Figure 1:**
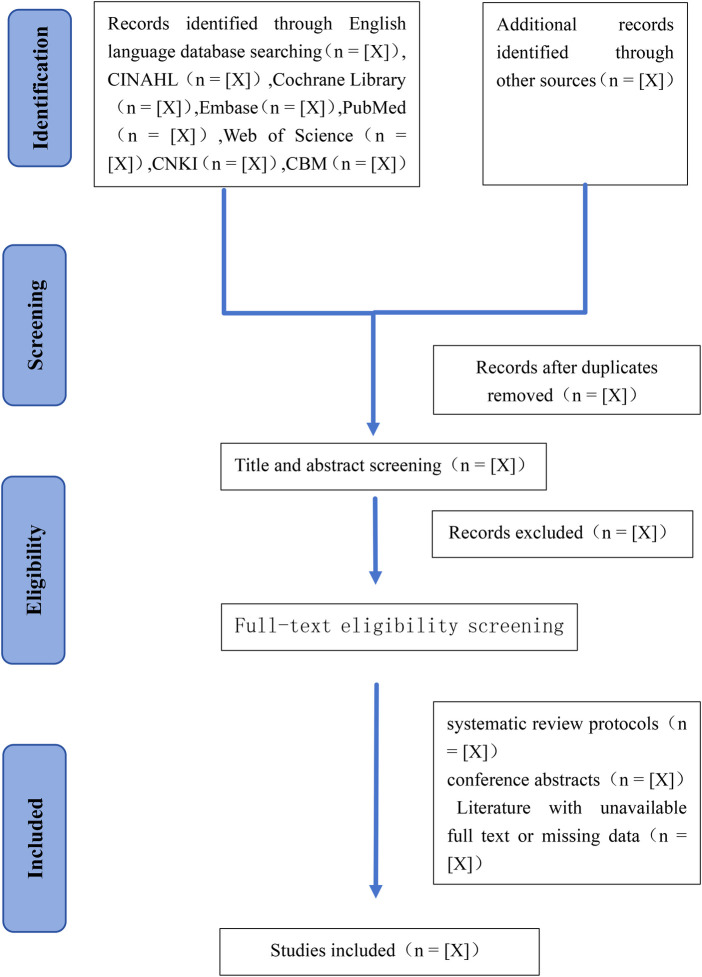
Planned literature screening flow diagram for the umbrella review. All counts are placeholders. Final numbers will be updated upon completion of the review.

A pilot calibration exercise will be conducted prior to formal screening, including at least 100 abstracts or 10% of all retrieved records (whichever is larger). The pilot will refine eligibility criteria interpretation and calibrate reviewer judgment. The final study selection flow will be presented in a PRISMA-O compliant flow diagram (Figure 1); all numerical values in the protocol version are placeholders.

#### 2.4.2 Data extraction

A standardized, pilot-tested data extraction form will be developed *a priori* and used by two independent reviewers (KS and YS). The full extraction form template is provided in Supplementary File 2. Extracted data will include:

1. SR characteristics: First author, publication year, country, funding source, conflict of interest statement, PROSPERO registration status, number and types of included primary studies, total sample size, search period, databases searched
2. Primary study inventory: Full list of primary studies included in each SR (required to quantify study overlap across reviews)
3. Participant characteristics: Age, gender, primary diagnosis, ICU type, baseline functional status
4. Intervention characteristics: Definition of ER, initiation timing, intervention domains, intensity classification (low/moderate/high), frequency, duration, delivery team composition
5. Comparator characteristics: Type of control intervention
6. Outcome measures: Outcomes reported, measurement tools, assessment time points, effect estimates with 95% CIs
7. Safety data: Whether adverse events were assessed, adverse event definitions, event classification system, and event rates

Missing or unclear data will be requested by emailing corresponding authors. If no response is received within 2 weeks, only available data will be used, and the impact of missing data will be discussed as a limitation.

### 2.5 Quality assessment and evidence grading

#### 2.5.1 Methodological quality of included SRs

Two independent reviewers (HL and AD) will assess the methodological quality of included SRs using the AMSTAR 2 tool (16 items). AMSTAR 2 is used exclusively to evaluate the conduct and reporting quality of the SRs themselves, not as a direct measure of risk of bias in the underlying primary evidence. Each item will be rated as Yes, Partial Yes, No, or No Information, and overall confidence in each SR will be classified as High, Moderate, Low, or Critically Low per standard AMSTAR 2 guidance.

We will extract and summarize risk-of-bias assessments as reported by the included SRs. *De novo* reassessment of risk of bias at the individual primary study level is not planned, and this is acknowledged as a limitation of the umbrella review design.

#### 2.5.2 Certainty of evidence grading

We will perform *de novo* GRADE assessments for each primary outcome, rather than extracting preexisting GRADE ratings from included SRs. Two independent reviewers will grade evidence certainty across five domains:

1. Risk of bias: Informed by AMSTAR 2 ratings and the risk of bias summary from the highest-quality SR for each outcome
2. Inconsistency: Evaluated based on heterogeneity in effect estimates and variability in intervention protocols
3. Indirectness: Evaluated based on differences in population, intervention, comparator, and outcome definitions
4. Imprecision: Evaluated based on sample size, confidence interval width, and event rates
5. Publication bias: Evaluated based on small-study effects and evidence of selective reporting

An additional downgrade will be applied if the corrected covered area (CCA) of primary study overlap exceeds 10%, indicating substantial dependence across evidence sources. Evidence certainty will be rated as High, Moderate, Low, or Very Low. Disagreements will be resolved by discussion or third-party adjudication.

### 2.6 Data synthesis and analysis

All synthesis will be conducted in accordance with PRISMA-O and PRIOR guidelines. Narrative synthesis is the primary analytical approach; quantitative synthesis will only be performed where methodologically appropriate.

#### 2.6.1 Quantification of study overlap

Prior to any quantitative synthesis, we will construct a citation matrix of all included primary studies across all SRs and calculate the corrected covered area (CCA) to quantify the degree of primary study overlap. Overlap will be categorized as slight (CCA <5%), moderate (5%–10%), or high (>10%).

#### 2.6.2 Narrative synthesis

We will first present a descriptive evidence map summarizing the characteristics, methodological quality, and key findings of all included SRs, organized by outcome domain, intervention type, and patient population. We will systematically compare consistency of effect estimates across reviews, and explore how differences in intervention protocols, population eligibility, and methodological quality may explain divergent findings.

#### 2.6.3 Quantitative synthesis

We will not pool effect estimates directly across SRs as independent units, as overlapping primary study samples would double-count participants and distort precision. Where feasible, we will extract individual primary study effect estimates from included SRs and construct a *de novo* meta-analysis with primary studies as the unit of analysis, using a random-effects (DerSimonian-Laird) model. This approach avoids duplicate counting by including each primary study only once per outcome.

Quantitative synthesis will only be performed if:

- There are ≥3 independent primary studies reporting the same outcome at the same time point
- Study overlap is manageable (CCA <10%)
- Intervention definitions and outcome measures are sufficiently homogeneous

For dichotomous outcomes, we will calculate pooled relative risks (RRs) with 95% CIs. For continuous outcomes, we will calculate pooled mean differences (MDs) or standardized mean differences (SMDs) with 95% CIs. Heterogeneity will be assessed using the *χ*^2^ test and I^2^ statistic.

#### 2.6.4 Subgroup and sensitivity analyses

Subgroup analyses will be conducted to explore sources of heterogeneity, where disaggregated data are available from included reviews:

- ER initiation timing: <24 h vs. 24–72 h post-ICU admission
- ER intervention type: physical-only vs. multidisciplinary (multi-domain)
- ER intensity: low (passive bed-based) vs. moderate (active sitting/standing) vs. high (ambulation/resistance)
- ER delivery team: nurse-led vs. therapist-led vs. multidisciplinary
- Patient population: mechanically ventilated vs. non-ventilated; ECMO vs. non-ECMO; elderly (≥65 years) vs. non-elderly (<65 years)
- SR methodological quality: high/moderate vs. low/critically low
- Publication year: ≤2018 vs. >2018

Sensitivity analyses will assess the robustness of findings:

- Excluding SRs rated as Low or Critically Low quality by AMSTAR 2
- Excluding SRs that include only observational study designs
- Restricting analysis to prospectively registered SRs
- Leave-one-out analysis for quantitative synthesis

#### 2.6.5 Publication bias assessment

Funnel plots and Egger's regression test will only be used for *de novo* meta-analyses of primary studies (where ≥10 primary studies are available for a given outcome). We will not perform publication bias testing at the SR level, as these methods are not validated for overview-level analyses and are unreliable when SRs share overlapping primary study samples. At the SR level, publication bias will be assessed narratively by comparing effect estimates from small and large SRs and evaluating evidence of selective outcome reporting.

## 3 Discussion

Despite growing consensus that early rehabilitation (ER) is a core component of high-quality critical care, real-world implementation remains suboptimal worldwide. Surveys from mainland China indicate that only 20%–30% of ICUs routinely deliver structured ER services, constrained by limited resources, insufficient trained personnel, safety concerns, and the absence of standardised clinical protocols (13); comparable barriers have been documented across other low- and middle-income settings. While accumulating systematic reviews have reported favourable effects of ER on functional outcomes and long-term quality of life (14, 15), evidence remains inconsistent regarding the safety and net benefit of ER in specific high-risk patient subgroups (16). Furthermore, there is no internationally agreed standard for ER content, intensity, or dosing, which limits reproducibility and cross-setting generalisability (17). These gaps in the evidence base highlight the need for a high-level evidence synthesis to map the current landscape, appraise methodological quality, and clarify where reliable conclusions can and cannot be drawn.

If the certainty of available evidence is sufficient, the findings of this umbrella review may inform evidence-based clinical decision-making, support the development of standardised ER protocols, and guide priority setting for future research. For clinicians, this synthesis may help identify which ER modalities and delivery models are supported by the most robust evidence. For healthcare policymakers, the graded evidence may inform the update of national and international critical care rehabilitation guidelines. For researchers, this work will explicitly identify evidence gaps—particularly in high-risk populations and long-term safety outcomes—to inform the design of future high-quality randomised controlled trials.

This overview has several predefined methodological strengths. First, it will systematically synthesise evidence on both the effectiveness and safety of ER across all four core domains (physical, respiratory, cognitive, psychological) and the full spectrum of adult ICU populations, providing a broader evidence map than existing narrower reviews. Second, we will apply rigorous and standardised methodological frameworks: the AMSTAR 2 tool for critical appraisal of included SRs, and the GRADE approach for grading the certainty of evidence for all key outcomes, in full adherence with PRISMA-O reporting guidelines. Third, we will include both English and Chinese literature from nine electronic databases, supplemented by manual reference checking, to capture a more comprehensive evidence base.

Several anticipated limitations should be acknowledged. First, included SRs will inevitably overlap in their primary study samples; this overlap may bias descriptive summaries and will be systematically documented and addressed as part of the analysis. Second, the reliability of our findings will be inherently dependent on the methodological quality of the included SRs; if most reviews are rated as low or critically low quality by AMSTAR 2, the overall evidence base will be considered weak. Third, substantial heterogeneity in ER protocols—including differences in initiation timing, intervention intensity, component modalities, and outcome measurement tools—is expected, which may preclude quantitative synthesis for some outcomes. Fourth, adverse event reporting across primary studies and SRs is known to be inconsistent and incomplete, which may limit the comprehensiveness of our safety synthesis. Fifth, we will restrict inclusion to studies published in English or Chinese, which introduces potential language bias and may miss relevant evidence published in other languages; additionally, there may be partial duplication of evidence across Chinese and English databases. Sixth, we will only include published, peer-reviewed SRs, which may introduce publication bias and will not capture ongoing or unpublished reviews (publication lag).

Importantly, all conclusions and clinical implications drawn from this umbrella review will be explicitly bounded by the certainty of the evidence as graded by GRADE. Where evidence is rated as low or very low certainty, we will refrain from making strong clinical recommendations and will instead emphasise the need for further high-quality primary research. This cautious approach ensures that our findings do not overstate the strength of the available evidence.

In summary,this protocol describes a planned overview of systematic reviews evaluating the effectiveness and safety of early rehabilitation in critically ill adult ICU patients. By appraising methodological quality, risk of bias, certainty of evidence, and overlap among primary studies, the overview may help identify consistent findings, unresolved questions, and priorities for future research. Clearer prespecification of eligibility criteria, search strategy, and synthesis methods will be essential to ensure that the final overview provides transparent and clinically useful evidence.
