# Supplementary material for: Early rehabilitation for critically ill adult patients: protocol for an umbrella review
Source: Front Rehabil Sci. 2026 Jul 22;7:1885510. doi: 10.3389/fresc.2026.1885510 (PMC13437619; doi:10.3389/fresc.2026.1885510)
Supplement: Supplementary file 1 [file Supplementaryfile1.docx]

### **Supplementary File 1:**

### **Full Search Strategies for All 9 Databases**

*Last updated: April 2026All strategies were developed following the PRESS (Peer Review of Electronic Search Strategies) standard.*

1. PubMed (MEDLINE)

#1 "Rehabilitation"[Mesh] OR "Exercise Therapy"[Mesh] OR "Early Ambulation"[Mesh] OR "Physical Therapy Modalities"[Mesh] OR "Respiratory Therapy"[Mesh]

#2 (early rehabilitat* OR early mobil* OR early mobility OR early exercise* OR early activ* OR ambulation OR mobilization OR physiotherapy OR physical therapy OR rehabilitation therap* OR exercise therap* OR cardiopulmonary rehabilitat*).ti,ab.

#3 "Critical Illness"[Mesh] OR "Intensive Care Units"[Mesh] OR "Critical Care"[Mesh]

#4 (critical ill* OR intensive care OR ICU OR critical care unit* OR mechanically ventilat* OR post intensive care syndrome OR PICS).ti,ab.

#5 "Systematic Review"[pt] OR "Meta-Analysis"[pt]

#6 (systematic review* OR meta-analys* OR umbrella review* OR overview of review* OR review of reviews OR evidence synthesis).ti,ab.

#7 #1 OR #2

#8 #3 OR #4

#9 #5 OR #6

#10 #7 AND #8 AND #9

#11 Filters: Humans, English, Chinese

2. Embase (Ovid)

#1 exp Rehabilitation/ OR exp Exercise therapy/ OR exp Early ambulation/ OR exp Physiotherapy/ OR exp Respiratory therapy/

#2 (early rehabilitat* OR early mobil* OR early mobility OR early exercise* OR early activ* OR ambulation OR mobilization OR physiotherapy OR physical therapy OR rehabilitation therap* OR exercise therap*).ti,ab.

#3 exp Critical illness/ OR exp Intensive care unit/ OR exp Critical care/

#4 (critical ill* OR intensive care OR ICU OR critical care unit* OR mechanically ventilat* OR post intensive care syndrome OR PICS).ti,ab.

#5 exp Systematic review/ OR exp Meta analysis/

#6 (systematic review* OR meta-analys* OR umbrella review* OR overview of review* OR review of reviews OR evidence synthesis).ti,ab.

#7 #1 OR #2

#8 #3 OR #4

#9 #5 OR #6

#10 #7 AND #8 AND #9

#11 Limit to human

#12 Limit to (English OR Chinese)

3. Cochrane Library

#1 MeSH descriptor: [Rehabilitation] explode all trees

#2 MeSH descriptor: [Exercise Therapy] explode all trees

#3 MeSH descriptor: [Early Ambulation] explode all trees

#4 (early rehabilitat* OR early mobil* OR early mobility OR early exercise* OR early activ* OR physiotherapy OR physical therapy):ti,ab,kw

#5 #1 OR #2 OR #3 OR #4

#6 MeSH descriptor: [Critical Illness] explode all trees

#7 MeSH descriptor: [Intensive Care Units] explode all trees

#8 (critical ill* OR intensive care OR ICU OR mechanically ventilat* OR PICS):ti,ab,kw

#9 #6 OR #7 OR #8

#10 "systematic review" OR "meta-analysis" OR "umbrella review" OR "overview of reviews":ti,ab,kw

#11 #5 AND #9 AND #10

4. CINAHL (EBSCOhost)

#1 (MH "Rehabilitation+") OR (MH "Exercise Therapy+") OR (MH "Early Ambulation+") OR (MH "Physical Therapy+")

#2 TI AB (early rehabilitat* OR early mobil* OR early mobility OR early exercise* OR early activ* OR physiotherapy OR physical therapy)

#3 S1 OR S2

#4 (MH "Critical Care+") OR (MH "Intensive Care Units+")

#5 TI AB (critical ill* OR intensive care OR ICU OR mechanically ventilat* OR post intensive care syndrome)

#6 S4 OR S5

#7 TI AB (systematic review* OR meta-analys* OR umbrella review* OR overview of review* OR evidence synthesis)

#8 S3 AND S6 AND S7

#9 Limit to Human

#10 Limit to English OR Chinese

5. Web of Science Core Collection

#1 TS=(early rehabilitat* OR early mobil* OR early mobility OR early exercise* OR early activ* OR physiotherapy OR physical therapy OR exercise therapy OR cardiopulmonary rehabilitation)

#2 TS=(critical ill* OR intensive care OR ICU OR critical care unit* OR mechanically ventilat* OR post intensive care syndrome OR PICS)

#3 TS=(systematic review* OR meta-analys* OR umbrella review* OR overview of review* OR review of reviews OR evidence synthesis)

#4 #1 AND #2 AND #3

#5 Document types: Review

#6 Languages: English, Chinese

#7 Human research

6. 中国知网（CNKI）

检索字段：主题=（早期康复 OR 早期活动 OR 早期运动 OR 早期功能锻炼 OR 心肺康复 OR 运动疗法）

AND 主题=（危重症 OR 重症监护 OR ICU OR 机械通气 OR 重症患者）

AND 主题=（系统评价 OR Meta分析 OR 荟萃分析 OR 伞状评价 OR 系统评价再评价）

检索范围：核心期刊、CSCD

时间：建库-2026.04.30

7. 中国生物医学文献数据库（CBM）

#1 主题词：康复/全部树/OR 运动疗法/全部树/OR 早期下床活动/全部树

#2 缺省：早期康复 OR 早期活动 OR 早期运动 OR 早期功能锻炼

#3 #1 OR #2

#4 主题词：危重病/全部树/OR 重症监护病房/全部树

#5 缺省：危重症 OR 重症监护 OR ICU OR 机械通气

#6 #4 OR #5

#7 缺省：系统评价 OR Meta分析 OR 荟萃分析 OR 系统评价再评价

#8 #3 AND #6 AND #7

8. 万方数据库

检索字段：主题

检索式：

(早期康复 OR 早期活动 OR 早期运动 OR 早期功能锻炼)

AND

(危重症 OR 重症监护 OR ICU OR 机械通气 OR 重症患者)

AND

(系统评价 OR Meta分析 OR 荟萃分析 OR 伞状评价)

文献类型：期刊论文、学位论文

时间：建库-2026.04.30

9. 维普数据库

检索字段：题名或关键词

检索式：

M=((早期康复 OR 早期活动 OR 早期运动 OR 早期功能锻炼) * (危重症 OR ICU OR 重症监护 OR 机械通气) * (系统评价 OR Meta分析 OR 荟萃分析))

专业检索限定：核心期刊

时间：建库-2026.04.30
