# Supplementary material for: Early rehabilitation for critically ill adult patients: protocol for an umbrella review
Source: Front Rehabil Sci. 2026 Jul 22;7:1885510. doi: 10.3389/fresc.2026.1885510 (PMC13437619; doi:10.3389/fresc.2026.1885510)
Supplement: Supplementary file 2 [file Supplementaryfile2.docx]

### **Supplementary File 2**

**Standardized Data Extraction Form Template**

*nstructions: Two independent reviewers will complete this form for each included systematic review. Discrepancies will be resolved via consensus or third-party adjudication.*

**Section A: Basic Information**

| **Field** | **Content** |
| --- | --- |
| Systematic review ID |  |
| First author |  |
| Publication year |  |
| Country of corresponding author |  |
| PROSPERO registration number (if any) |  |
| Funding source |  |
| Conflict of interest statement (Yes/No/Not stated) |  |
| Extracted by |  |
| Checked by |  |
| Date of extraction |  |

**Section B: SR Characteristics**

| **Field** | **Content** |
| --- | --- |
| Number of included primary studies |  |
| Types of included study designs (RCT / NRCT / observational) |  |
| Total sample size (all participants) |  |
| Databases searched |  |
| Search time range |  |
| Quality assessment tool used in the SR |  |

**Section C: Primary Study Inventory**

List full citation (first author, year) of all primary studies included in this SR. This information will be used to calculate corrected covered area (CCA) of study overlap.

1.

2.

3.

**Section D: Participant Characteristics**

| **Field** | **Content** |
| --- | --- |
| Mean age (range) |  |
| Proportion of male participants (%) |  |
| Primary diagnosis (e.g., general medical, surgical, neurological, ECMO) |  |
| ICU type (general / cardiac / neuro / mixed) |  |
| Minimum ICU length of stay required |  |
| Baseline functional status |  |

**Section E: Intervention Characteristics**

| **Field** | **Content** |
| --- | --- |
| Definition of "early rehabilitation" used in the SR |  |
| Initiation timing (hours/days post-ICU admission) |  |
| Core domains included (physical / respiratory / cognitive / psychological) |  |
| Intervention intensity (low / moderate / high) |  |
| Frequency (sessions per day / per week) |  |
| Average session duration |  |
| Total intervention duration |  |
| Delivery team (nurse-led / therapist-led / multidisciplinary) |  |
| Passive interventions only? (Yes / No / Mixed) |  |

**Section F: Comparator Characteristics**

| **Field** | **Content** |
| --- | --- |
| Type of control group (standard care / delayed rehabilitation / lower intensity) |  |
| Description of control intervention |  |

**Section G: Outcome Measures**

| **Outcome Domain** | **Measurement Tool** | **Time Point** | **Effect Estimate (RR/MD/SMD)** | **95% CI** |
| --- | --- | --- | --- | --- |
| Physical function |  |  |  |  |
| Cognitive function |  |  |  |  |
| Psychological function |  |  |  |  |
| ICU LOS |  |  |  |  |
| Hospital LOS |  |  |  |  |
| Mechanical ventilation duration |  |  |  |  |
| Mortality (28/90-day) |  |  |  |  |
| ICU readmission rate |  |  |  |  |

**Section H: Safety Data**

| **Field** | **Content** |
| --- | --- |
| Were adverse events systematically reported? (Yes / No / Partially) |  |
| Definition of adverse events used |  |
| Total number of adverse events (intervention group) |  |
| Total number of adverse events (control group) |  |
| Severe adverse events listed |  |
| Minor adverse events listed |  |

**Section I: Methodological Quality**

| **AMSTAR 2 Item** | **Rating (Yes / Partial Yes / No / No Information)** |
| --- | --- |
| Item 1: Research question and inclusion criteria |  |
| Item 2: Protocol registration |  |
| ... (all 16 items) |  |
| **Overall AMSTAR 2 confidence rating** | High / Moderate / Low / Critically Low |
