# Supplementary material for: Early rehabilitation for critically ill adult patients: protocol for an umbrella review
Source: Front Rehabil Sci. 2026 Jul 22;7:1885510. doi: 10.3389/fresc.2026.1885510 (PMC13437619; doi:10.3389/fresc.2026.1885510)
Supplement: Supplementary file 3 [file Supplementaryfile3.docx]

### Supplementary File 3

### Completed PRISMA-P 2015 Checklist

Protocol title: Early rehabilitation for critically ill adult patients: a protocol for an umbrella review (overview of systematic reviews)

| **Item No.** | **PRISMA-P 2015 Item Description** | **Status** | **Location in Manuscript** |
| --- | --- | --- | --- |
| 1 | Identify the report as a systematic review protocol | Done | Title page, Section 2.1 |
| 2 | Provide a structured summary of the protocol, including objectives, methods, and planned analyses | Done | Abstract section |
| 3 | Describe the rationale for the review in the context of existing knowledge | Done | Section 1 (Introduction) |
| 4 | State the primary and secondary review objectives explicitly | Done | Section 1, final paragraph |
| 5 | Specify study characteristics as inclusion/exclusion criteria (PICO framework) | Done | Section 2.2 (Eligibility Criteria) |
| 6 | List all information sources (databases, registers, grey literature) and date ranges | Done | Section 2.3.1 |
| 7 | Present the full search strategy for at least one database | Done | Table 1 + Supplementary File 1 |
| 8 | Describe processes for managing records and data | Done | Section 2.4.1 (EndNote + Rayyan workflow) |
| 9 | Describe the study selection process (dual independent screening, disagreement resolution) | Done | Section 2.4.1 |
| 10 | Describe the data collection process (dual independent extraction, missing data handling) | Done | Section 2.4.2 |
| 11 | List and prioritize all planned outcomes, specify measurement tools and time points | Done | Section 2.2.3 |
| 12 | Specify the tool(s) for assessing risk of bias/methodological quality | Done | Section 2.5.1 (AMSTAR 2) |
| 13 | Describe the planned data synthesis approach (narrative/quantitative) and heterogeneity assessment | Done | Section 2.6 |
| 14 | If meta-analysis is planned, specify the model and effect measures | Done | Section 2.6.3 (de novo primary-study level meta-analysis) |
| 15 | Describe plans for grading the certainty of evidence | Done | Section 2.5.2 (GRADE framework) |
| 16 | Disclose potential conflicts of interest for all review authors | Done | Declarations section |
| 17 | Describe sources of funding for the review | Done | Declarations section (Funding) |
| 18 | Provide the systematic review registration number and registration platform | Done | Abstract + Section 2.1 (PROSPERO CRD420250651183) |
